# Supplementary material for: 5-Hydroxymaltol Derived from Beetroot Juice through Lactobacillus Fermentation Suppresses Inflammatory Effect and Oxidant Stress via Regulating NF-kB, MAPKs Pathway and NRF2/HO-1 Expression
Source: Antioxidants (Basel). 2021 Aug 23;10(8):1324. doi: 10.3390/antiox10081324 (PMC8389248; doi:10.3390/antiox10081324)
Supplement: Supplementary file 1 [file antioxidants-10-01324-s001.zip › antioxidants-1273984-supplementary.pdf]

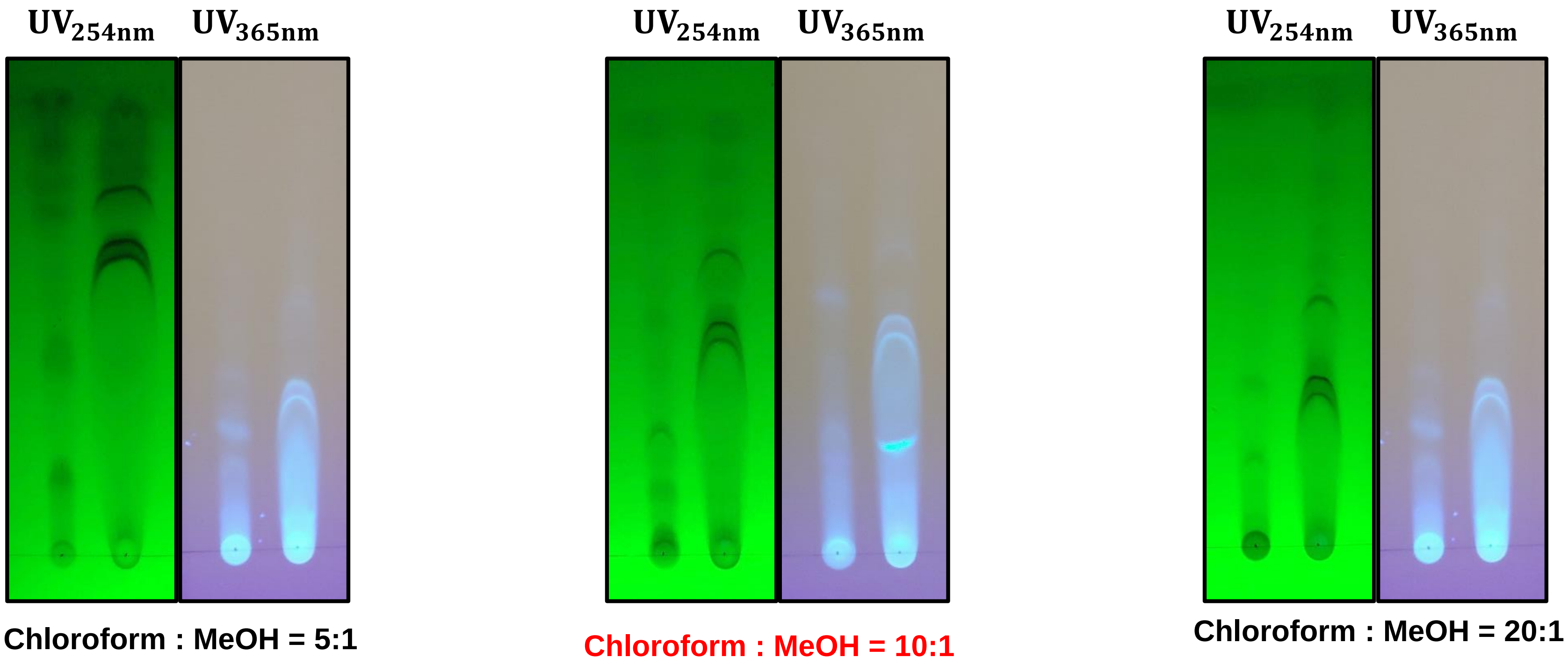

Supplementary Figure S1. Purification procedure of antioxidant from beet fermentation by *Lactobacillus* using ethylacetate extraction (fermentated solution: ethylacetate=1:1)

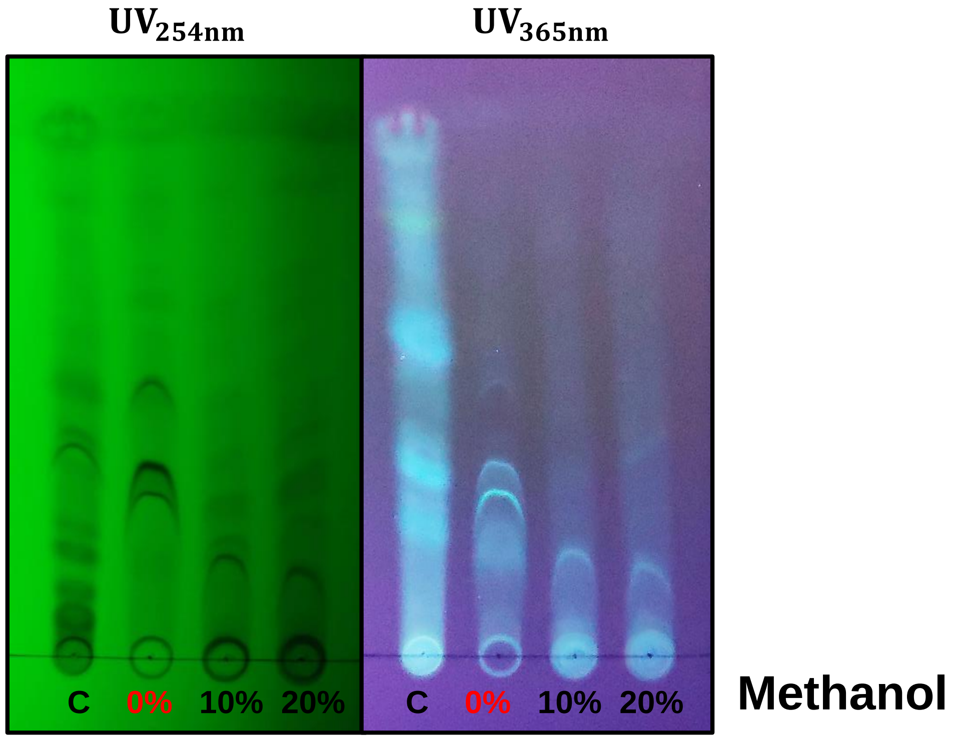

Supplementary Figure S2. Purification procedure of antioxidant from beet fermentation by *Lactobacillus* using ODS (C-18) chromatography. TLC plate analysis of the purified sample. Active fraction; 0% methanol.

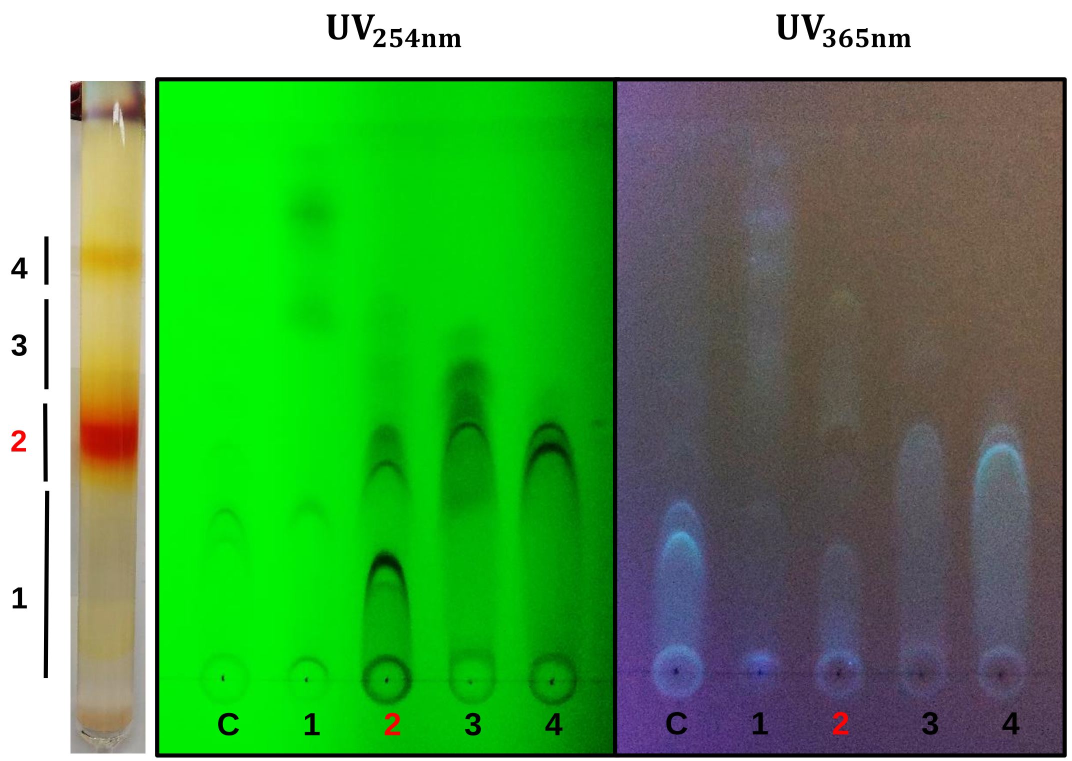

Supplementary Figure S3. Purification procedure of antioxidant from beet fermentation by *Lactobacillus* using Silica gel chromatography eluted with $CHCl_3$: MeOH (10:1). TLC plate analysis of the purified sample. Active fraction; #2.

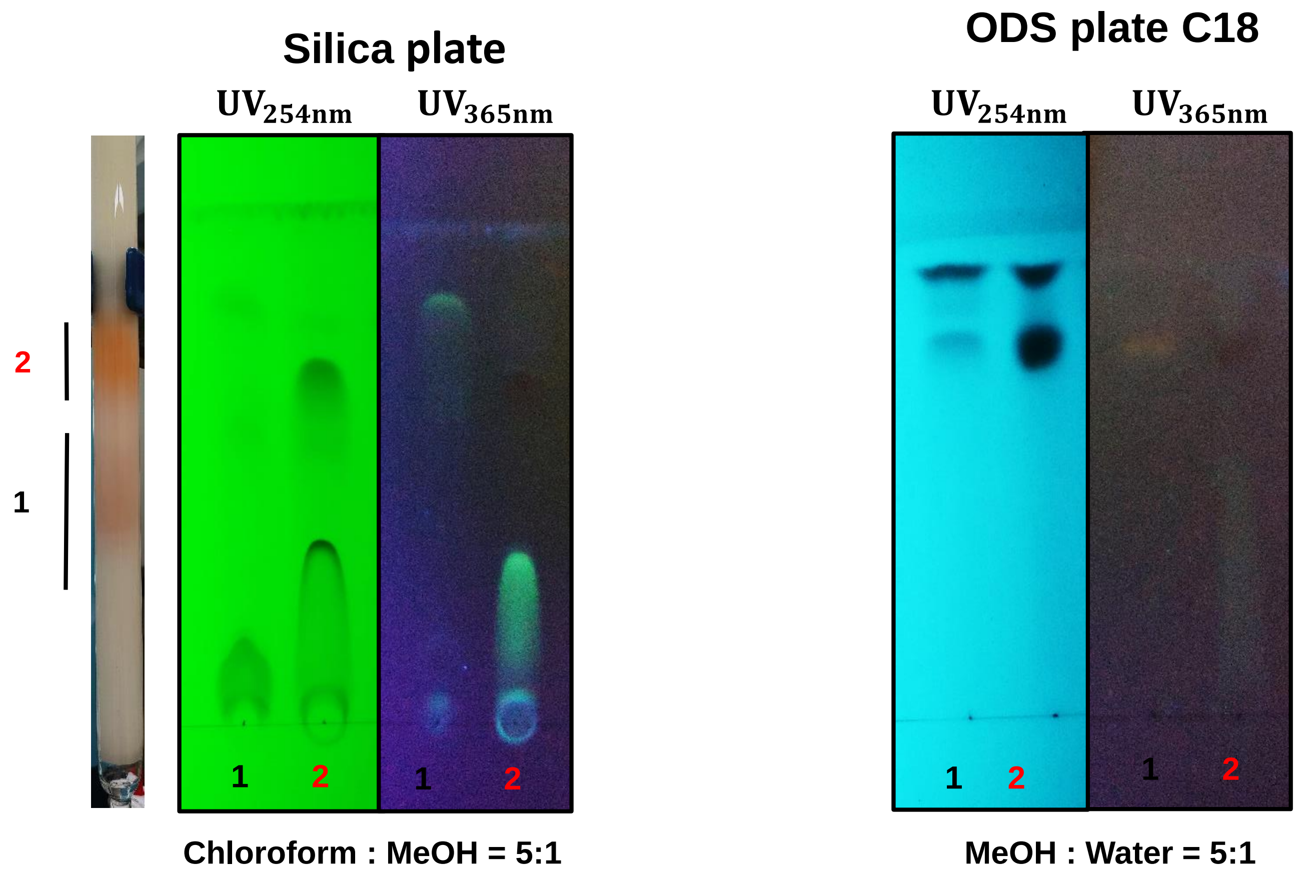

Supplementary Figure S4. Purification procedure of antioxidant from beet fermentation by *Lactobacillus* using Sephadex G-10 with water. TLC plate analysis of the purified sample. Active fraction; #2.

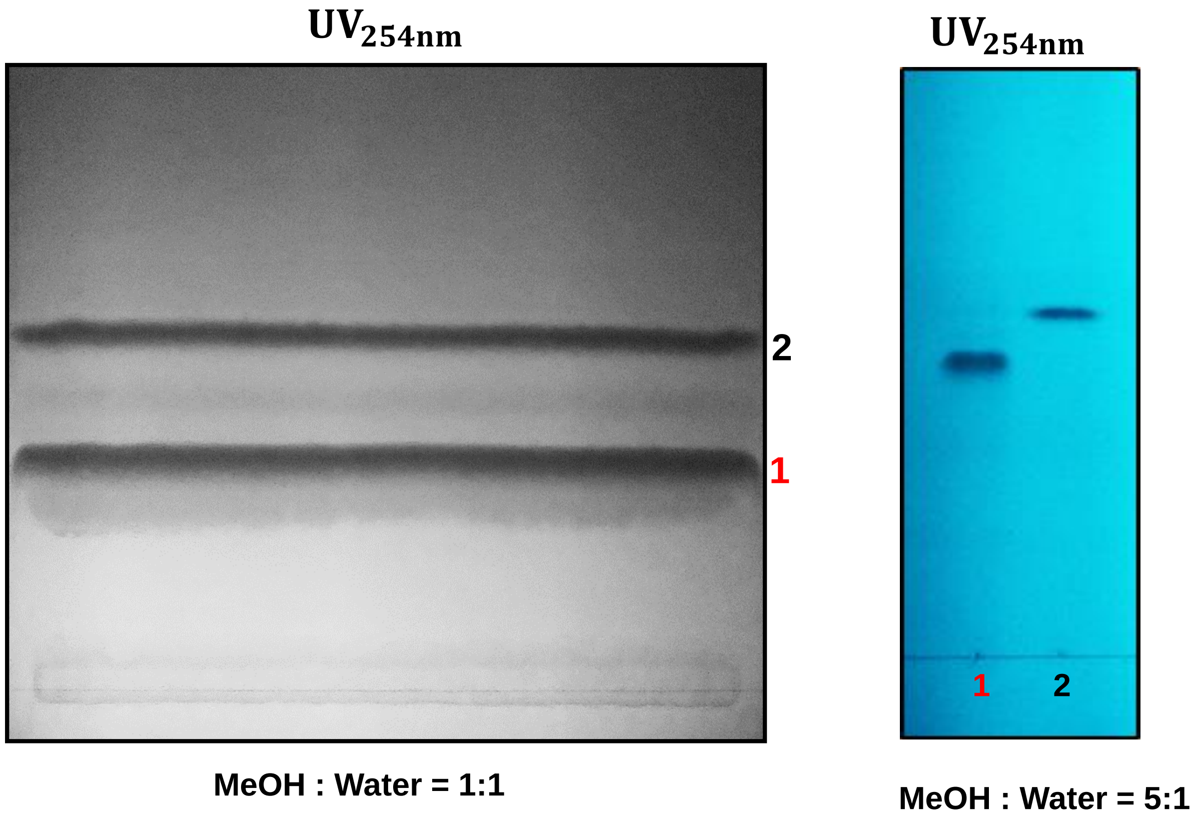

Supplementary Figure S5. Purification procedure of antioxidant from beet fermentation by *Lactobacillus* using C-18 Prep TLC. TLC plate analysis of the purified sample. Active fraction; #1.

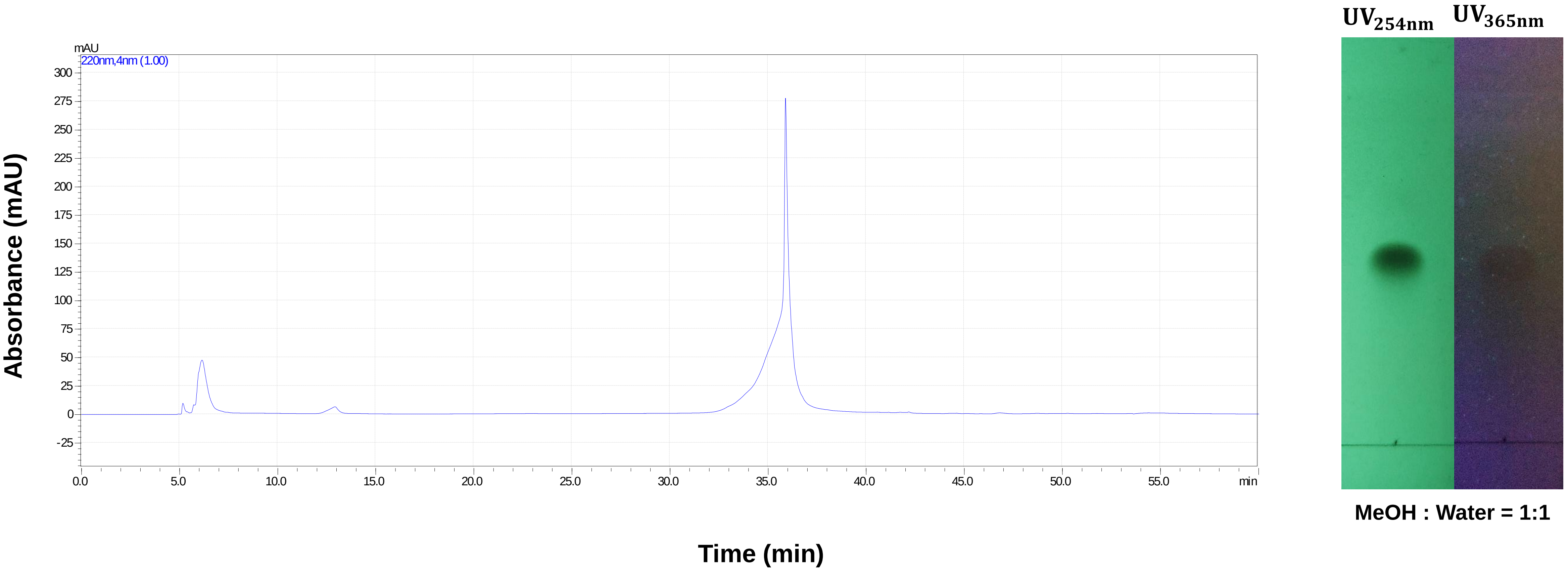

Supplementary Figure S6. Purification procedure of antioxidant from beet fermentation by *Lactobacillus* using Prep HPLC with ACN.

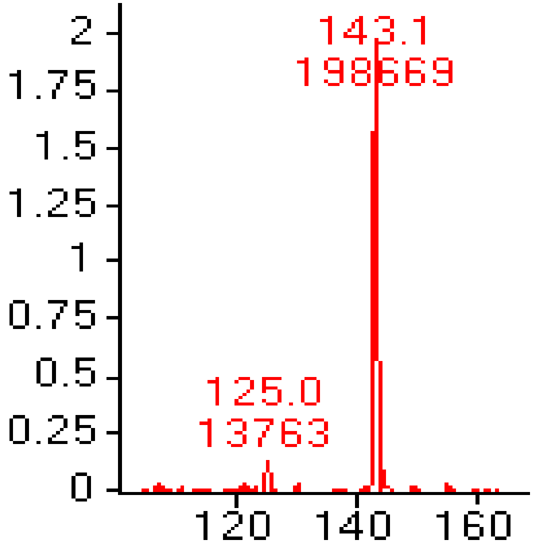

Supplementary Figure S7. ESI-mass spectrometry of the purified sample.

^{1}H NMR

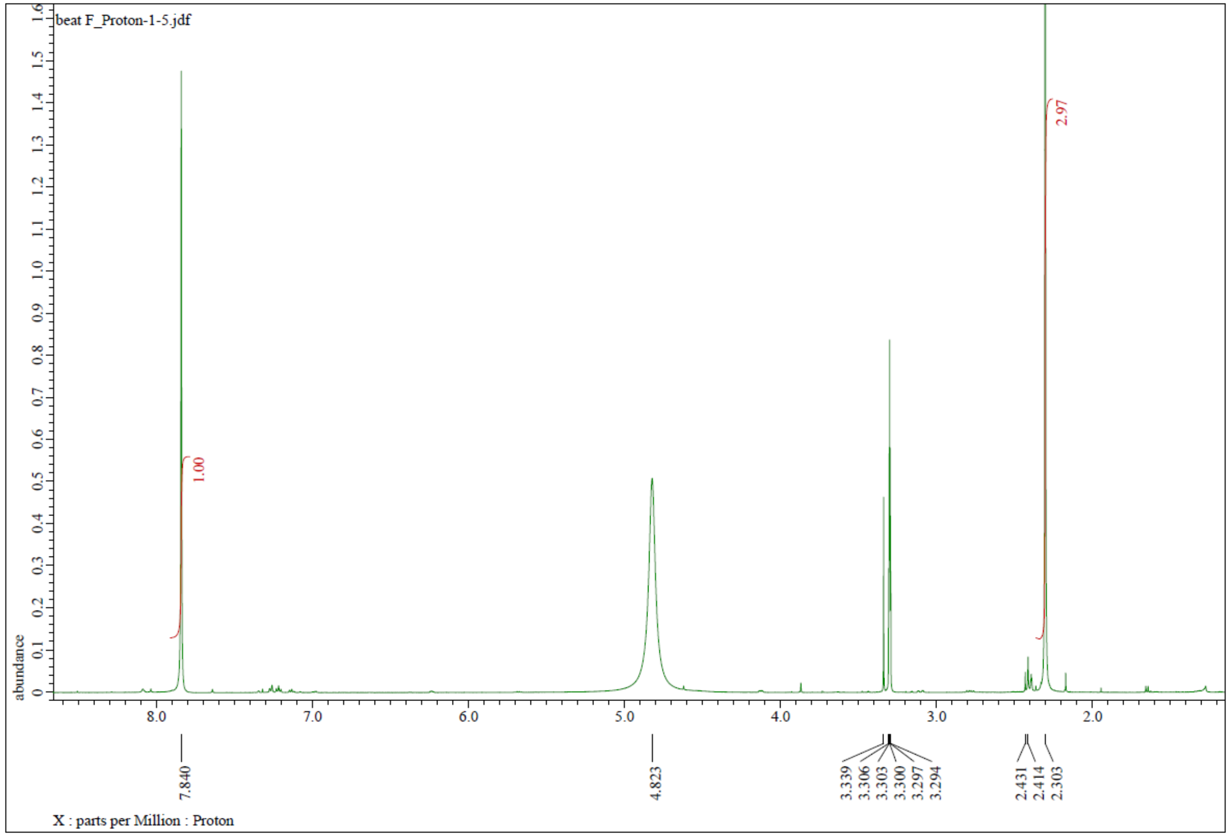

^{13}C NMR

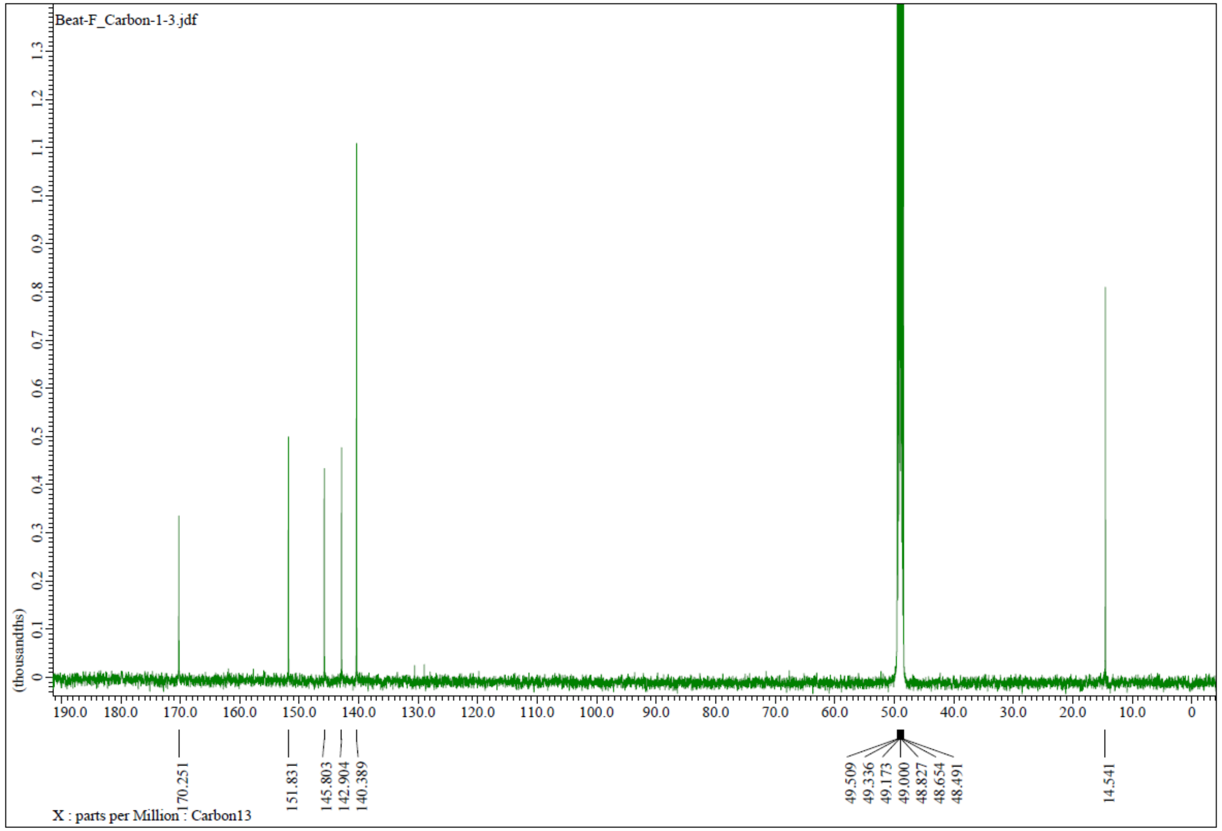

Supplementary Figure S8. ^{1}H NMR and ^{13}C NMR spectra of the purified sample.

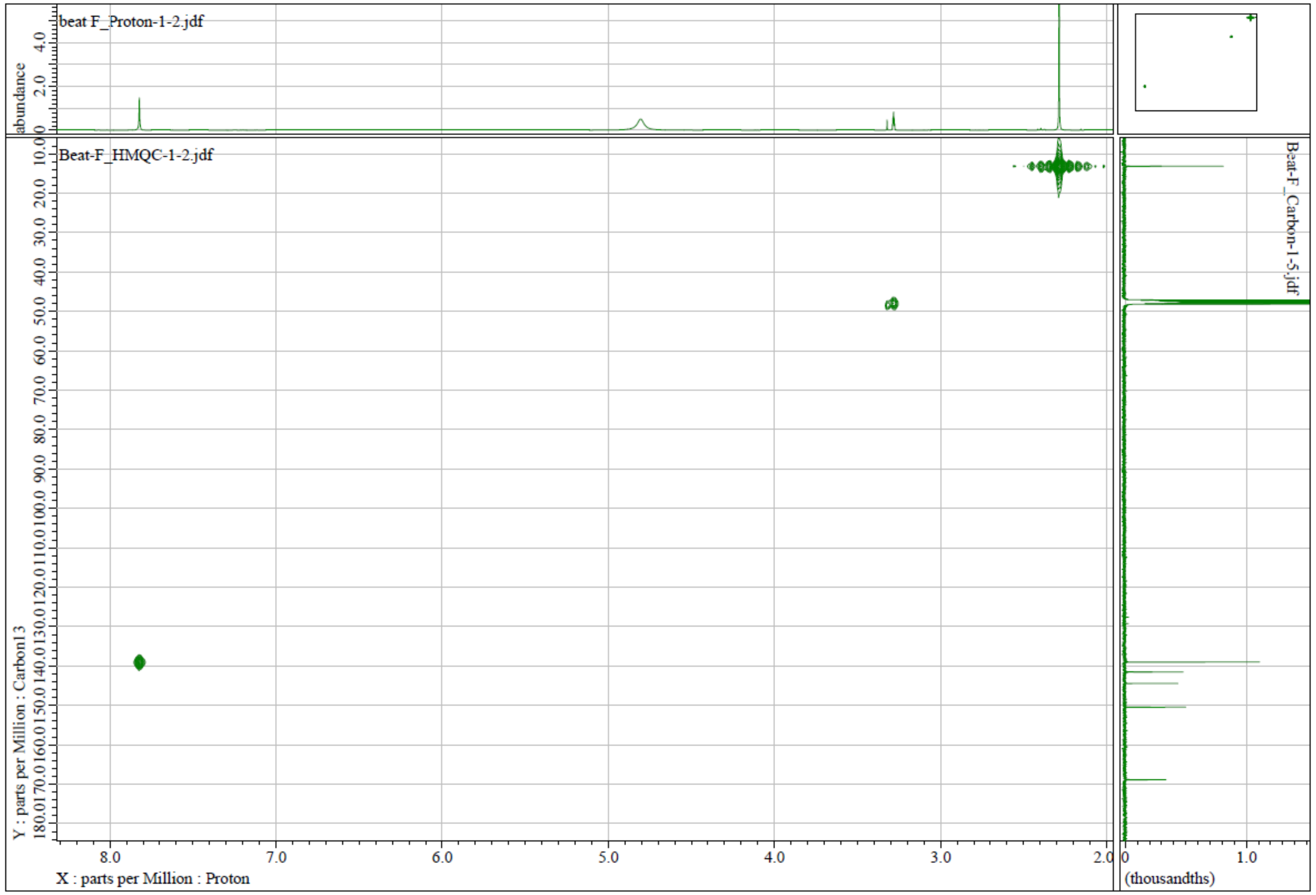

Supplementary Figure S9. HMQC spectrum of the purified sample

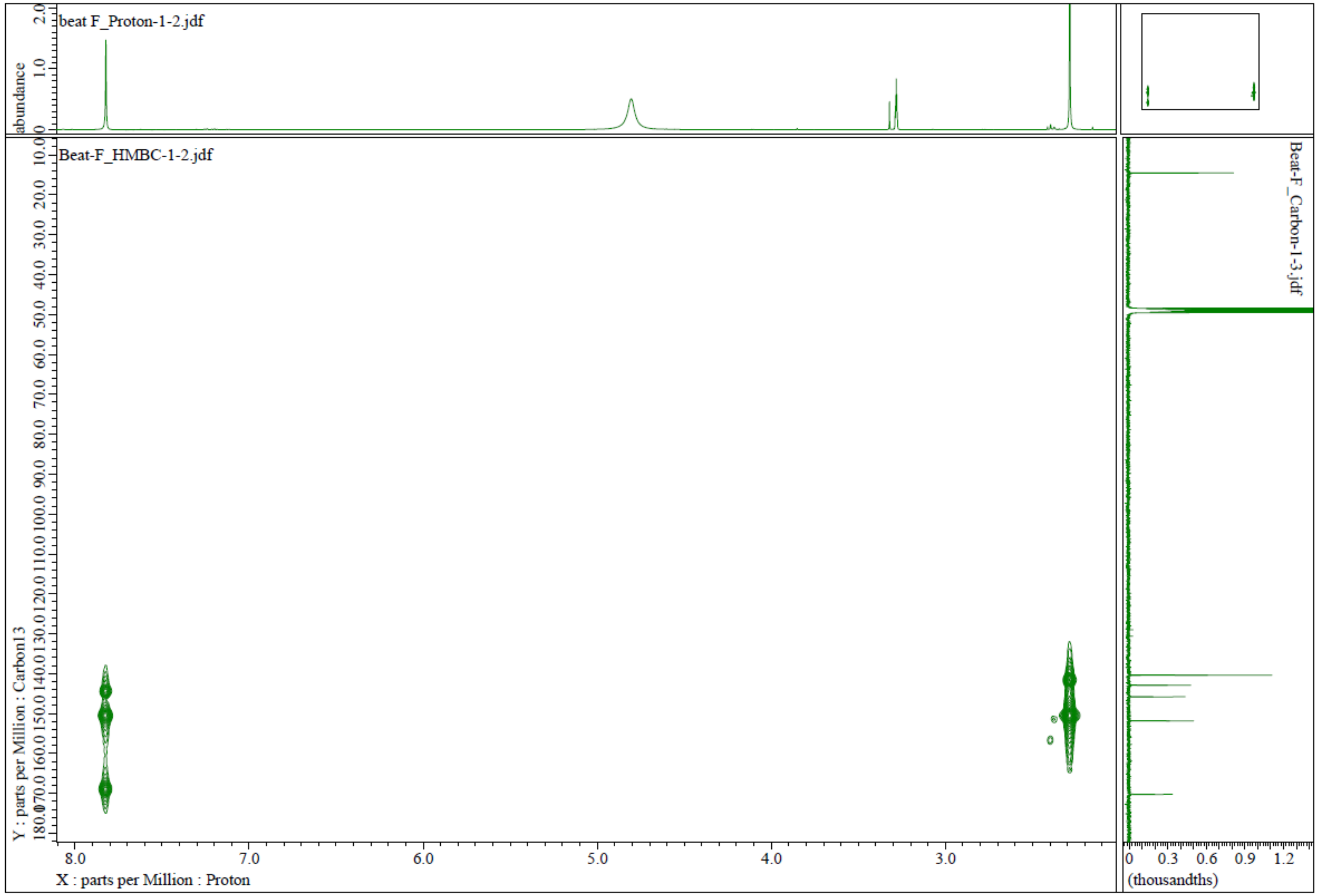

Supplementary Figure S10. HMBC spectrum of the purified sample.

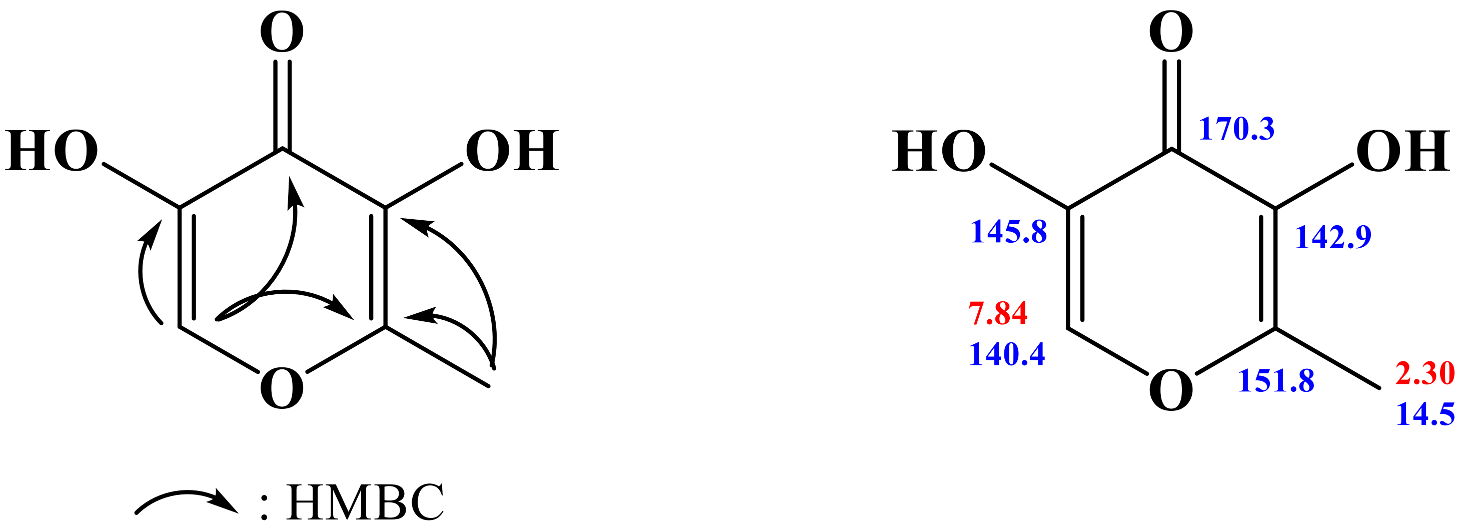

Supplementary Figure S11. HMBC correlations and ^{1}H (red) and ^{13}C (blue) peaks assignments of the purified sample.
